# Assessing the assessment: a scoping review of the mode of patient-reported outcome assessment in solid cancer clinical trials

**DOI:** 10.1007/s11136-026-04179-y

**Published:** 2026-03-01

**Authors:** Niclas Hubel, Daniela Krepper, Lotte van der Weijst, Abigirl Machingura, Claudia Seidl, Samuel M. Vorbach, Michelle Veil, Johannes M. Giesinger, Monika Sztankay, Scottie Kern, Deborah Fitzsimmons, Dagmara Kuliś, Madeline Pe, Bernhard Holzner, Jens Lehmann

**Affiliations:** 1 Health Outcomes Research Unit, University Hospital of Psychiatry II, Medical University of Innsbruck, Innrain 52, 6020 Innsbruck, Austria; 2 Quality of Life Department, European Organisation for Research and Treatment of Cancer, Avenue Mounierlaan 83/11, 1200 Brussels, Belgium; 3 Department of Radiation Oncology, Medical University of Innsbruck, Anichstraße 35, 6020 Innsbruck, Austria; 4 Critical Path Institute, 1840 E River Rd, Suite 100, Tucson, AZ USA; 5 Swansea Centre for Health Economics, Swansea University, Singleton Park, Swansea, SA2 8PP Wales, UK

**Keywords:** Patient-reported outcomes, Cancer, Mode of assessment, Clinical trials

## Abstract

**Purpose:**

Transparent reporting of how patient-reported outcomes (PROs) are collected is essential to ensure reproducible and interpretable data. Different modes of assessment may affect data quality and feasibility, yet their use in cancer trials is poorly described. Electronic PRO (ePRO) assessment may improve data quality and enable active review, but it is unclear how often different modes of assessment like ePRO assessment are used and in which trials.

**Methods:**

We systematically searched PubMed for randomized controlled trials (published 2019–2023) that used cancer-specific PRO measures in patients with the six most common solid cancers. Trial characteristics, PRO reporting practices, and evidence of active review of PRO data were summarized descriptively. Univariate logistic regression was used to examine predictors of (1) reporting the mode of PRO assessment and (2) use of ePROs exclusively.

**Results:**

Of 9331 references screened, 296 trials were included in the analysis. 135 (45.6%) reported the mode of PRO assessment: paper (51.9%), ePRO (20.7%), mixed modes (24.4%). Trials were more likely to report the mode of assessment if they were industry-sponsored (OR = 2.00, 95%, *p* = .005), or had larger sample sizes (OR = 1.11, 95%, *p* < .001). ePRO assessment exclusively was used more in recently registered trials (OR = 1.41, *p* < .001) and in industry-sponsored trials (OR = 8.38, *p* < .001). Active, in-stream review of PRO results was reported in 2.0% of trials.

**Conclusion:**

Despite clear guidelines, reporting of the mode of PRO assessment remains inadequate, and active review of PRO data is uncommon. Strengthening transparency and using PROs more actively within trials could enhance patient-centered cancer research.

**Supplementary Information:**

The online version contains supplementary material available at 10.1007/s11136-026-04179-y.

## Abstract (plain language summary)

This study looked at how information from patients about their symptoms and quality of life is collected in cancer trials. These patient-reported outcomes are important because they show how treatments affect people’s daily lives, not just medical results. To be useful, this information needs to be collected and reported clearly so that others can trust and learn from it. We reviewed 296 cancer trials published between 2019 and 2023. The aim was to investigate whether the trials explained how they gathered questionnaires, and if the results were checked during the trial to support patient care. We found that less than half of the trials clearly reported how the questionnaires were collected. When it was reported, paper questionnaires were most common, although electronic tools are being used more often in newer and industry-sponsored studies. Only a very small number of trials reported that they actively reviewed questionnaire results from individual patients while the trial was still running. The findings show that in cancer trials, it is often not clearly explained how patient information is collected, even though this is recommended by guidelines. Better reporting of how questionnaires are assessed and used as part of cancer trials is needed to make trial methodology more transparent.

**Supplementary Information:**

The online version contains supplementary material available at 10.1007/s11136-026-04179-y.

## Introduction

Patient-reported outcomes (PROs) are playing an increasingly important role in cancer clinical trials. As self-reported reflections of patients’ perspectives, PROs capture vital information on symptoms, quality of life, functional status, and the burden of treatment, factors that are essential to understanding the full impact of cancer therapies beyond traditional clinical endpoints [1, 2]. Regulatory bodies such as the U.S. Food and Drug Administration (FDA) [3] and the European Medicines Agency (EMA) [4] have recognized this growing importance, incorporating PRO data into decision-making processes for drug approval and labeling claims [3–6]. Future steps were further concretized in two recent joint publications by various interest groups advocating for greater utilization of PROs in the regulatory environment [5, 6].

Given this relevance, it is critical to ensure that PRO-based endpoints are held to high methodological and reporting standards. These outcomes must be meaningful, replicable, and assessed using clearly described procedures. To support such rigor, specific guidelines have been developed, most notably the SPIRIT-PRO extension for trial protocols and the CONSORT-PRO extension for trial reporting [7, 8]. Both emphasize the importance of specifying how, when, and where PRO data are collected, which are critical to reproducing methods and evaluating PRO data quality. While these aspects are often overlooked, empirical research has shown that the timing and setting of PRO assessments can meaningfully influence the results [9, 10]. Specifically, Giesinger et al. [9] observed significant differences in perceived treatment burden between assessments conducted on the day of chemotherapy admission versus assessments conducted one week later at home, whereas Shiroiwa et al. [10] identified systematic differences between electronic remote and on-site paper-based assessments. Such findings underscore the rationale for the detailed recommendations made in the SPIRIT-PRO and CONSORT-PRO guidelines.

Further, the mode of how PRO data are collected can vary. Historically, paper-based questionnaires were the standard, but over time, electronic methods, using provisioned devices, web-based solutions, or bring-your-own-device (BYOD) solutions, have been introduced. A growing body of evidence, summarized in two meta-analyses, supports the comparability of different PRO data collection modes [11, 12], provided that best practices for implementation and instrument migration are followed [13]. The authors stress the consistent demonstration of the equivalence of validity of electronically administered measures [12] as well as the high cross-mode retest reliability [11]. Regulatory guidance, including from the FDA, accepts multiple modes of administration as long as comparability and usability are demonstrated [14]. The most recent recommendations from an International Society for Pharmacoeconomics and Outcomes Research (ISPOR) task force provide clear criteria for determining when electronically administered PROs can be considered comparable to their paper counterparts [15]. Building on this, electronic PRO (ePRO) data collection offers several potential advantages over traditional methods. Several sources suggest potential advantages of ePRO systems over paper-based assessment, including improved data quality, such as fewer missing or invalid responses [16]. Reviews and commentaries further note that ePRO platforms allow real-time monitoring of completion and the use of automated reminders, which may support more timely and complete data capture [17, 18]. The presence of an electronic audit trail also facilitates traceable, time-stamped records and system validation procedures that support data integrity and regulatory compliance [19]. In addition, diary-based studies indicate that electronic data capture may be associated with higher patient compliance, particularly for very frequent (e.g., daily) assessments [20]. Organizations such as the Center for Medical Technology Policy [21] and regulatory bodies, including the FDA [14] and EMA [4, 22], have acknowledged the benefits of using electronic methods for PRO data collection. However, challenges remain, including the risk of sampling bias [16, 23] and limited digital literacy [23, 24] in certain populations, usability issues [23], as well as uncertainties about how ePRO systems are integrated into routine trial workflows [17]. Systematic evidence on how frequently different modes of PRO assessment are used in clinical trials, whether paper, electronic, or hybrid, remains limited. Moreover, there is little systematic evidence about how different trial characteristics like trial size, blinding, or location of PRO data assessment are associated with the mode of PRO assessment.

Finally, ePRO assessment theoretically allows for real-time monitoring of patient-reported symptoms and proactive clinical management [25]. Based on literature highlighting the benefits of using PROs to inform individual patients’ care or improving clinical teams’ awareness of patients health status [26–28] it has been suggested to also use PROs collected as part of clinical trials for patients immediate management or for trial documentation purposes (e.g., to inform adverse event documentation) [25]. It remains unclear, however, how often such active, in-stream review is reported in protocols or used in practice. Recent evidence from ovarian cancer studies suggests that active review of PRO results is still uncommon in cancer trials [29].

This scoping review aims to examine how patient-reported outcome measures (PROMs) are collected and reported in randomized controlled trials involving patients with solid cancers. Specifically, we describe how and where PROs are assessed, and how clearly these methods are reported following relevant SPIRIT-PRO [8] and CONSORT-PRO [7] reporting items. We further explore whether certain trial characteristics, such as sponsorship, sample size, or phase, are associated with more transparent reporting or the use of electronic assessment, as these factors may influence the feasibility and implementation of different modes. Finally, we investigate whether PRO data were actively reviewed by trial personnel during the course of the trial.

## Methods

The review was reported according to the PRISMA-ScR (Preferred Reporting Items for Systematic Reviews and Meta-Analyses extension for Scoping Reviews) 2020 checklist [30] (see Supplementary Material 1).

A systematic search of MedLine (PubMed interface) was conducted to identify RCTs using PROs as an endpoint in the six most prevalent solid tumor types (lung, breast, prostate, colorectal, bladder, gynecological) [31], published between January 2019 and November 2023. Only trials investigating biomedical interventions and utilizing PRO instruments developed by either the European Organisation for Research and Treatment of Cancer (EORTC) or the Functional Assessment of Chronic Illness Therapy (FACIT) measurement system were included. We focused on EORTC or FACIT questionnaires, as these are by far the most used tools in cancer clinical trials [32]. A comprehensive description of the eligibility criteria and the full search strategy is provided in the published protocol [33].

A pool of six reviewers participated in the screening and data extraction process. All steps were executed in DistillerSR [34], which was used to coordinate independent reviews, track discrepancies, and document resolutions throughout the process. Two reviewers independently screened abstracts and full-text articles to determine eligibility. Discrepancies were first resolved through discussion, and if consensus could not be reached, a third reviewer was consulted to reach a final decision. Following the selection process, trials were matched using their registration number or study acronym, as data extraction was conducted at the trial level rather than per individual publication. This approach enabled the comprehensive inclusion of information from associated publications. Additionally, trial protocols were included if available. For data charting, reviewer pairs independently extracted information for each included study within DistillerSR to ensure accuracy and consistency. Any disagreements were discussed within the pair and resolved by consensus, with final decisions documented in the software.

Extracted variables included key trial characteristics such as industry sponsorship, trial organization involvement (not necessarily sponsorship), year of first trial registration, blinding, trial phase, disease stage, type of treatment evaluated, control arm design, and the sample size of the intention-to-treat (ITT) population. With respect to the PRO endpoints, we documented whether PROs were designated as pre-defined trial endpoints (either primary, secondary or exploratory). The subsequent part of the data extraction form was informed by relevant EQUATOR guidelines (i.e., SPIRIT [35] and the SPIRIT-PRO extension [8] for protocols, CONSORT [36] and CONSORT-PRO extension [7] for published trials). Information on the PRO data management and assessment encompassed the mode(s) of assessment (specifying which modes were used and, if multiple modes were used, if evidence for comparability was cited [15]), the assessment setting (field-based vs. site-based assessment), and whether PRO data were actively reviewed by trial personnel or healthcare professionals during the trial (e.g., a site nurse or a doctor reviewed individual patients PRO scores).

### Statistical analysis

Trial characteristics were summarized using descriptive statistics. Categorical variables are presented as frequencies with corresponding percentages, while continuous variables are reported as means with standard deviations.

We conducted univariate logistic regression analyses to examine trial characteristics as predictors of two binary outcomes:

1. *Reporting of the PRO mode of assessment*: Each covariate was individually tested to assess if it predicted whether a trial disclosed their mode of PRO assessment (reported vs not-reported).
2. *Exclusive electronic PRO assessment*: Models identified factors associated with trials solely assessing PRO electronically, compared to all other modes (e.g. hybrid or paper assessment methods)

Predictors included in the univariate analyses were: Date of trial registration, disease stage, cancer site, industry sponsoring, availability of a study protocol, sample size, trial organisation involvement, trial phase, and whether the PRO endpoint was defined as primary, secondary, or exploratory (including not defined). The selection of predictor variables was guided by our published protocol [30] and by theoretical considerations related to trial design and resource availability. For instance, industry sponsorship may facilitate electronic data capture through greater infrastructure and regulatory expectations, whereas smaller or academic trials may rely on paper-based methods due to resource constraints. Similarly, larger and later-phase trials may have greater operational capacity and standardized processes supporting electronic data collection. Given the limited empirical evidence on these associations, our analyses were exploratory and aimed to identify potential patterns for future research rather than causal relationships. Model coefficients are expressed as odds ratios (ORs), each with 95% CIs and an α level of .05. Calculations were done using R version 4.3.1 [37].

## Results

**Fig. 1:**
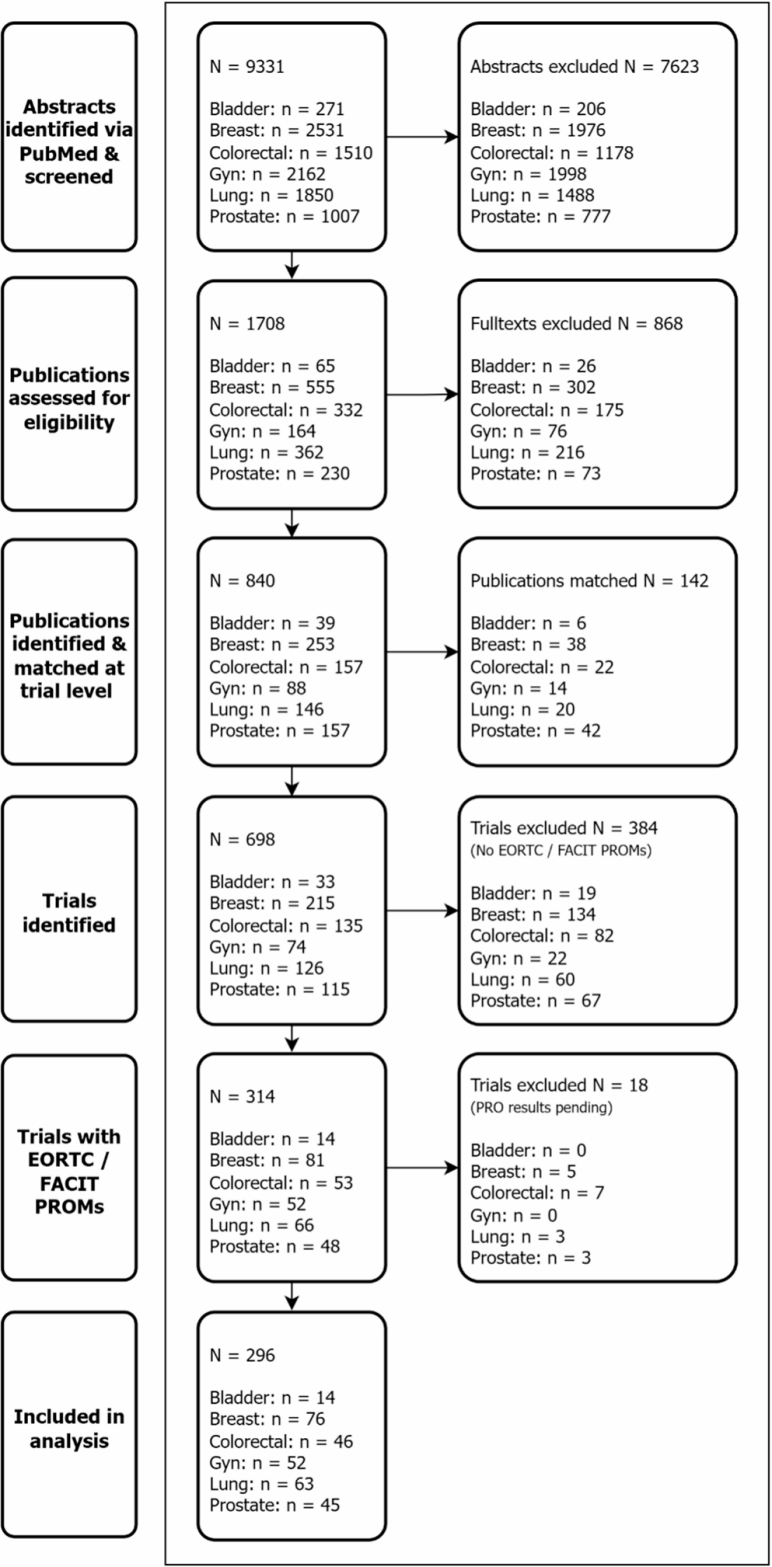
PRISMA flowchart

Our initial literature search yielded 9331 references. After title and abstract screening for eligibility, we included 1708 publications in the full-text review (Fig. 1). The resulting 840 articles were matched on the trial level, resulting in 698 trials. We excluded trials without results from EORTC (201/698, 28.8% of all trials using any PRO endpoint) and FACIT (105/698, 15.0%) PROMs. A final number of 296 trials were included in the analysis. Figure 1 depicts the PRISMA-ScR flowchart.

### Trial characteristics and reporting of PRO mode of assessment

Trials were registered between 2001 and 2022 (median: 2014), with the last publications ranging from 2019 to 2023 (Table 1). Breast cancer was the most common cancer type (n = 76, 25.7%), and most studies were Phase III trials (n = 182, 61.5%). Industry sponsorship was identified in 35.8% of trials (n = 106), while the remaining trials were either academically sponsored or did not specify their sponsor. Separately, 29.7% of trials (n = 88) involved a trial organization in the conduct or coordination of the study. The most frequently involved organizations were the National Cancer Institute (n = 33, 11.1%) and NRG Oncology (n = 8, 2.7%). PROs were predominantly used as secondary endpoints (n = 220, 74.6%).

**Table 1:** Trial characteristics

Variable	N = 296
Year of trial registration (N, %)	
2018–2022	28 (9.5)
2013–2017	142 (48.0)
2008–2012	68 (23.0)
Before 2008	32 (10.8)
No registration date	26 (8.8)
Cancer site (N, %)	
Bladder cancer	14 (4.7)
Breast cancer	76 (25.7)
Colorectal cancer	46 (15.5)
Gynecological cancer	52 (17.6)
Lung cancer	63 (21.3)
Prostate cancer	45 (15.2)
Trial organization involvement (N, %)	
Yes	88 (29.7)
No	208 (70.3)
Industry sponsor (N, %)	
Yes	106 (35.8)
No	190 (64.2)
Trial phase (N, %)	
II	61 (20.6)
III	182 (61.5)
IV	4 (1.4)
Not reported	49 (16.6)
Treatment evaluated (multiple could apply; N, %)	
Targeted therapy	83 (28.0)
Chemotherapy	80 (27.0)
Other treatment	60 (20.4)
Radiotherapy	38 (12.8)
Surgery	37 (12.5)
Hormonal therapy	31 (10.5)
Immunotherapy	23 (7.8)
Control condition (N, %)	
Active comparator	233 (78.77)
Placebo controlled	63 (21.3)
PRO endpoint (N, %)	
Primary	27 (9.2)
Secondary	220 (74.6)
Exploratory (including not defined)	48 (16.3)
PROMs used (multiple could apply; N, %)	
EORTC questionnaires	201 (67.9)
FACIT questionnaires	105 (35.5)
ITT sample size (mean (SD))	543.06 (757.17)
PRO sample size (mean (SD)) ^1^	447.66 (576.93)
Blinding (N, %)	
No blinding, open-label	191 (64.6)
Yes, double-blinded	70 (23.7)
Yes, single-blinded	6 (2.0)
Not reported	31 (9.8)
Disease stage (N, %)	
Mainly metastatic/advanced	138 (46.6)
Mainly non-metastatic/local	101 (34.1)
Both	39 (13.2)
Not reported	18 (6.1)
Protocol available (N, %)	
Yes	161 (54.4)
No	135 (45.6)

PRO = Patient-reported Outcome; ITT = Intention to treat

^1^Defined as the number of patients with completed PRO data at baseline; some trials included PRO assessments as a sub-study rather than as part of the main trial

Assessment location was reported in 153/296 trials (51.7%) and took place field-based only (6/296, 2%), site-based (91/296, 30.7%), or both (56/296, 18.9%).

A total of 135/296 (45.6%) trials reported the mode of PROM assessment (Table 2). Specifically, 67/296 (22.6%) reported it only in the protocol, 36/296 (12.2%) only in the publication, and 32/296 (10.8%) in both publication and protocol. Out of the 135 trials reporting the mode of assessment, 70/135 (51.9%) used paper only, and 28/135 (20.7%) used electronic PRO assessment only. Among trials mixing different modes of assessment (33/135), 13/135 (9.6%) used ePRO and paper, 19/135 (14.1%) used paper and non-automated telephone interviews, and one trial (0.7%) mixed paper and interviewer administration. Evidence for comparability between mixed modes of assessment was provided in just one of the 33 trials (3%) using multiple modes.

**Table 2:** Patient-reported outcome measure assessment characteristics in trials

Variable	
Location of PRO data collection (N, %)	N = 296
Field-based (ie, remote only)	6 (2.0)
Site-based (ie, at the study sites only)	91 (30.7)
Both site-based and field-based	56 (18.9)
Not reported	143 (48.3)
Mode of assessment (among trials that reported the mode; N, %)	N = 135
Paper only	70 (51.9)
Electronic PRO assessment only	28 (20.7)
Mixed-mode assessment (paper and non-automated telephone interviews)	19 (14.1)
Mixed-mode assessment (ePRO and paper)	13 (9.6)
Other (eg, non-automated telephone scripts)	5 (3.7)
Specified electronic PRO assessment mode (multiple could apply; N, %)	N = 41
Provisioned device	22 (53.7)
Electronic assessment mode not specified further	12 (29.3)
Website	6 (14.6)
‘Bring your own device’ (BYOD)	2 (4.9)

PRO = Patient-reported Outcome; ITT = Intention to treat

When PROs were assessed electronically, they were primarily assessed on a provisioned device (22/41, 53.7%) or via an unspecified modality (12/41, 29.3%). The complete table of evidence is given in Supplementary Materials 2.

### Active in-stream review

A total of six trials (2.0%) reported active review of PRO data by investigators or site staff, as specified in their protocols (Table S1). In five trials, this review was limited to identifying and documenting potential adverse events. One trial instructed treating clinicians to review PRO responses after toxicity ratings to identify symptoms requiring clinical attention and initiate supportive care if necessary. Among the six trials, two used ePRO, two used paper-based assessment, and two used a mixed approach.

### Trial characteristics and mode of assessment

Univariate logistic regression analyses were conducted to examine predictors of whether a trial reported the PRO mode of assessment (Table 3). The odds of reporting the mode were 2 times higher when the trial was sponsored by industry (OR = 2.00, 95% CI [1.24, 3.25], *p* = .005). When a protocol was available, the odds of reporting the mode were 9.49 times higher (OR = 9.49, 95% CI [5.57, 16.66], *p* < .001). For every additional 100 participants in the ITT sample, the odds of reporting the mode of assessment increased by 11% (OR = 1.11, 95% CI [1.06, 1.18], *p* < .001). Finally, compared to phase II trials, phase III trials were associated with 2.21 times higher odds of reporting the mode of assessment (OR = 2.21, 95% CI [1.22, 4.07], *p* = .009).

**Table 3:** Univariate regression models for reporting the mode of patient-reported outcome assessment

Variable^1^	Univariate			
	OR	95% CI		*p*
		LL	UL	
Date of trial registration (per year)	1.00	0.94	1.06	0.952
Advanced cancer population vs other populations ^2^	1.18	0.75	1.87	0.474
Cancer site (Reference: Bladder)				
Breast	3.87	1.10	18.09	0.050
Colorectal	2.82	0.76	13.70	0.148
Gynecological	3.14	0.86	15.10	0.106
Lung	3.55	1.00	16.82	0.070
Prostate	2.44	0.65	11.92	0.214
Industry sponsor vs. no industry sponsor	**2.00**	**1.24**	**3.25**	**0.005**
Protocol available vs. no protocol available	**9.49**	**5.57**	**16.66**	**< .001**
Sample size ITT (per 100 patients)	**1.11**	**1.06**	**1.18**	**< .001**
Trial organisation involvement vs. no involvement	1.37	0.83	2.26	0.215
Trial phase (Reference: II)				
III	**2.21**	**1.22**	**4.07**	**0.009**
Other phase (‘not reported’ or phase IV)^3^	0.52	0.22	1.17	0.121
PRO endpoint (Reference: Primary)				
Secondary	1.44	0.64	3.40	0.384
Exploratory (including not defined)	1.63	0.63	4.37	0.318

Significant (p < 0.05) variables in bold. Dependent variable: Mode of assessment reported (yes = 1); CI: Confidence interval; LL: lower level; UL: upper level

^1^Constants for univariate models are not shown

^2^Binary; at least 80% of the trial sample consists of patients with mainly metastatic or advanced cancer

^3^Trial phase IV and ‘not reported’ combined into a single category as both most likely contain most post market trials and the number of phase IV trials was too low to calculate a distinct category

Univariate logistic regression analyses were conducted to examine predictors of whether a trial used exclusively ePRO assessment (Table 4). For each additional year of trial registration, the odds of using ePRO were 1.41 times higher (OR = 1.41, 95% CI [1.22, 1.68], *p* < .001). From zero uses in trials registered before 2009 to a maximum of 4/10 (40.0%) trials registered in 2018. Trials involving patients with advanced cancer were associated with 3.72 times higher odds of using ePRO (OR = 3.72, 95% CI [1.72, 8.45], *p* = .001), indicating 272% higher odds of using ePRO. The odds of using ePRO were 8.38 times higher in trials sponsored by industry (OR = 8.38, 95% CI [3.67, 20.83], *p* < .001), and 3.87 times higher when a protocol was available (OR = 3.87, 95% CI [1.24, 17.09], *p* = .037). Notably, only one trial involving a trial organisation used ePRO (no OR calculated). Finally, phase III trials were associated with 12.04 times higher odds of using ePRO compared to phase II trials (OR = 12.04, 95% CI [2.40, 219.27], *p* = .017).

**Table 4:** Univariate regression models for ePRO assessment

Variable^1^	Univariate			
	OR	95% CI		*p*
		*LL*	*UL*	
Date of trial registration (per year)	**1.41**	**1.22**	**1.68**	** < .001**
Advanced cancer population vs other populations ^2^	**3.72**	**1.72**	**8.45**	**0.001**
Cancer site (Reference: Bladder)				
Breast	0.69	0.06	15.78	0.771
Colorectal	0.50	0.04	12.34	0.607
Gynecological	0.40	0.03	9.81	0.495
Lung	1.88	0.16	42.84	0.622
Prostate	1.27	0.10	30.47	0.855
Industry sponsor vs. no industry sponsor	**8.38**	**3.67**	**20.83**	** < .001**
Protocol available vs. no protocol available	**3.87**	**1.24**	**17.09**	**0.037**
Sample size ITT (per 100 patients)	1.00	0.95	1.03	0.807
Trial organisation involvement vs. no involvement^3^	–	–	–	–
Trial phase III	**12.04**	**2.40**	**219.27**	**0.017**
PRO endpoint (Reference: Exploratory (including not defined))				
Primary ^3^	–	–	–	–
Secondary	0.39	0.15	1.04	0.053
EORTC questionnaires used	2.01	0.91	4.77	0.096
FACIT questionnaires used	0.54	0.23	1.18	0.134
Site-based assessment vs. other modes	2.06	0.98	4.38	0.057

Significant (p < 0.05) variables in bold. Dependent variable: Mode of assessment reported (yes = 1)

CI: Confidence interval; LL: lower level; UL: upper level

^1^Constants for univariate models are not shown

^2^Binary; at least 80% of the trial sample consists of patients with mainly metastatic or advanced cancer

^3^Could not be analyzed due to too few cases: Only a single trial with trial organisation involvement used ePRO; No trials using PROs as primary outcome used ePRO

## Discussion

Our review revealed that less than half of all trials reported the mode of PRO assessment, with paper methods remaining the most common, and exclusively electronic assessment remained rare. Reporting was more likely in industry-sponsored trials, those with available protocols, larger sample sizes, and phase III design. The use of exclusively electronic PRO methods increased over time and was more common in industry-sponsored trials and those involving patients with advanced cancer. Active review of PRO results during the trial was almost never reported.

### Insufficient reporting of the mode of assessment of patient-reported outcomes

Despite clear guidance from SPIRIT-PRO and CONSORT-PRO, fewer than half of the trials (45.6%) in our review reported the mode of PRO assessment, highlighting a persistent and concerning gap in reporting standards. While this represents an improvement compared to what we know from earlier periods (2007–2011: 16% [38]), our review covered trials published between 2019 and 2023, well after the introduction of CONSORT-PRO in 2013. The lack of progress is echoed by Efficace et al. [39], who found that the mode of assessment was reported in around 22 percent of publications. Compared to that review, we found higher rates of reporting the mode of assessment, likely because we also included available protocols in our review. When considering only information from publications, we found a similar rate of 23% of trials reporting the mode. This highlights the lack of progress in reporting and reinforces the importance of publicly sharing protocols [40, 41].

Additionally, we observed that evidence of comparability between mixed modes of administration was reported in only one of 33 trials using multiple modes, underlining a broader issue of methodological transparency. Although it is established that paper and electronic PRO assessments are comparable when appropriate migration procedures are followed [13] or evidence for comparability is cited [15], we find that these assumptions are rarely supported by trial-level documentation. Reporting the mode of assessment is essential for transparency, reproducibility, and the interpretation of PRO results. This is especially important when modes differ in characteristics likely to influence responses, such as self-administered questionnaires versus interviewer-based assessments. As CONSORT-PRO notes, patients may respond differently when completing measures in private versus in a telephone interview [7]. Moreover, including such information requires minimal space or could be done in the supplementary materials, making arguments about word count limitations difficult to justify.

### Use and reporting of electronic patient-reported outcome assessment

The underreporting of the PRO mode of assessment also limits understanding of how ePRO data collection methods are used in clinical trials. While there has been long-standing enthusiasm for ePROs [18, 42] and our findings show increasing adoption, particularly in recent and industry-sponsored trials, overall use of ePROs remains modest in our trial sample. Paper-based methods likely remain the default, especially in non-industry-sponsored trials, suggesting that many trials not reporting the mode used paper. As a result, the observed 30.3% ePRO use (alone or in mixed modes) may overestimate actual adoption.

Electronic data collection was more frequent in industry-sponsored trials, possibly due to greater resources and regulatory expectations around auditability. However, despite the growing feasibility of BYOD strategies, their adoption remains limited, even when considering that most trials in our review were planned several years ago. A lack of public case studies where BYOD data supported regulatory approvals may contribute to sponsor hesitancy in adopting or reporting BYOD approaches [43] and therefore, be a byproduct of the inadequate mode of PRO assessment reporting we observed in our review.

Moreover, while field-based assessments are possible, most trials in our review still relied on site-based data collection. We found little evidence of decentralized strategies or participant-centered flexibility in assessment modes. Trials rarely implemented multiple modes of administration, and even in mixed-mode studies, variation was mainly due to pragmatic follow-up via mail or telephone rather than intentional patient choice. This reinforces concerns raised in recent literature that decentralized trial methods and participant-tailored approaches are still the exception rather than the rule [44].

### Active review of patient-reported outcome data during trials

We found almost no evidence that PRO data were actively reviewed for trial monitoring or for clinical care during the course of the trial. Among trials that actively reviewed PROs during the trial, they were primarily used to identify and document adverse events. While trial-level instructions for this may exist outside the main protocol, especially for industry-sponsored trials, our review found little indication of such supplementary guidance. A 2018 regulatory perspectives paper authored by representatives from major U.S. regulatory and oversight bodies acknowledged the debate about whether PRO data should be reviewed during a trial [45]. The authors emphasized that PROs are not considered safety data because they lack clinical interpretation. They noted that PROs could be reviewed during a trial, for example to support adverse event ratings, but that such review is not required. This cautious stance, recognizing the possibility of in-trial review without mandating it, may help explain why active review has so far gained little traction in oncology trials.

Electronic systems make real-time scoring and review feasible, yet this potential remains largely untapped. Active monitoring could improve both patient care and trial data quality [25, 46]. Some trialists might worry that such active review could introduce bias but ignoring available PRO data may also introduce inconsistency. Unstructured review of questionnaires likely occurs at individual sites, creating undocumented variability across centers [47]. Several barriers may explain the limited use of active PRO review, although these remain largely speculative due to limited empirical evidence [see [48] for more in-depth discussion). Implementing such processes would require a cultural and operational shift from current trial practices, change that might be met with resistance in the highly regulated context of clinical research. Additional resources and infrastructure may be needed to integrate real-time PRO monitoring into existing systems, increasing cost and logistical complexity. Moreover, the expected benefits have not yet been clearly quantified, and PROs are still frequently viewed as secondary rather than core trial data, potentially reducing the incentive for active use. Whether PRO data are reviewed in real time also reflects a broader trial design question. Active clinical use of PROs aligns more with pragmatic trials focused on real-world benefit, especially as PRO monitoring systems are increasingly used in routine clinical practice [49]. Passive electronic capture without clinical response fits an explanatory approach prioritizing internal validity. These trial-level decisions have ethical and methodological implications. If patients contribute data, there is a responsibility to use it meaningfully, ideally not only in publications but also to support care when appropriate.

### Limitations

First, our search relied on trial publications mentioning PROs, which may have excluded trials with PROs listed only in protocols. However, such endpoints are typically reported in publications, so most relevant trials were likely captured. Second, we limited our search to a single database and to studies published between 2019 and 2023 to manage workload, which may have led to some eligible trials being missed. Still, our aim was to reflect the most recent trial reporting practices.

Third, to enhance methodological consistency and feasibility, we confined our analysis to trials employing the two most extensively validated and widely implemented measurement systems in oncology (EORTC and FACIT). While this decision may have excluded some otherwise eligible trials utilizing other validated instruments, the absence of significant differences in reporting quality between EORTC- and FACIT-based studies supports the generalizability of our conclusions (data not shown). Nonetheless, this restriction may have led to a modest overestimation of reporting quality, as non-validated or self-developed instruments might be associated with less rigorous reporting practices.

Fourth, our regression analysis was exploratory in nature. We used univariable models to examine associations between trial characteristics and both the reporting of the mode of PRO assessment and the use of ePRO. While some of these characteristics may be correlated, our aim was to highlight potential patterns rather than to establish causal or independent effects. Hence, these associations should be interpreted cautiously, and future research is needed.

Finally, restricting our regression analysis to trials that reported some information on their PRO mode of administration may introduce sampling bias by favoring studies with more complete reporting. However, the direction of this potential bias remains uncertain, as it could, for instance, either overestimate or underestimate the use of electronic data capture.

## Conclusion

This review highlights persistent gaps in the reporting of PRO data collection methods in cancer trials, despite long-standing guidelines. Transparent reporting of the mode of PRO assessment is essential for reproducibility, data interpretation, and systematic evidence generation. Despite much enthusiasm for ePRO data collection and growing use over time, it remains relatively scarce. To make ethical and full use of the data that patients provide, the research field should commit not only to better reporting practices but also to actively using PRO data.

## Supplementary Information

Below is the link to the electronic supplementary material.

Supplementary Material 1

Supplementary Material 2

Supplementary Material 3

## Data Availability

The complete data from the review are available in Supplementary Material 3.

## References

[1] Anderson-Hanley, C., Sherman, M. L., Riggs, R., Agocha, V. B., & Compas, B. E. (2003). Neuropsychological effects of treatments for adults with cancer: A meta-analysis and review of the literature. *Journal of the International Neuropsychological Society,**9*(7), 967–982. 14738279 10.1017/S1355617703970019

[2] LeBlanc, T. W., & Abernethy, A. P. (2017). Patient-reported outcomes in cancer care—Hearing the patient voice at greater volume. *Nature Reviews. Clinical Oncology,**14*(12), 763–772. 28975931 10.1038/nrclinonc.2017.153

[3] U.S. Department of Health and Human Services FDA Center for Drug Evaluation and Research. Core patient-reported outcomes in cancer clinical trials: guidance for industry [Internet]. 2024. Available from: https://www.fda.gov/regulatory-information/search-fda-guidance-documents/core-patient-reported-outcomes-cancer-clinical-trials

[4] European Medicines Agency. Appendix 2 to the guideline on the evaluation of anticancer medicinal products in man - The use of patient-reported outcome (PRO) measures in oncology studies [Internet]. 2016 [cited 2023 Mar 17]. Available from: https://www.ema.europa.eu/en/documents/other/appendix-2-guideline-evaluation-anticancer-medicinal-products-man_en.pdf

[5] Pe, M., Voltz-Girolt, C., Bell, J., Bhatnagar, V., Bogaerts, J., Booth, C., et al. (2025). Using patient-reported outcomes and health-related quality of life data in regulatory decisions on cancer treatment: Highlights from an EMA-EORTC workshop. *The Lancet Oncology,**26*(6), 687–690. 40245904 10.1016/S1470-2045(25)00150-0

[6] Pignatti, F., Mol, P., Quinten, C., Postmus, D., Schiel, A., Sasseville, M., et al. (2025). Use of patient-reported outcomes to inform symptom and functional outcomes in cancer drug regulatory decisions: Challenges and future directions. *The Lancet Oncology,**26*(6), 664–666. 40245905 10.1016/S1470-2045(25)00151-2

[7] Calvert, M., Blazeby, J., Altman, D. G., Revicki, D. A., Moher, D., Brundage, M. D., et al. (2013). Reporting of patient-reported outcomes in randomized trials: The CONSORT PRO Extension. *JAMA,**309*(8), 814. 23443445 10.1001/jama.2013.879

[8] Calvert, M., Kyte, D., Mercieca-Bebber, R., Slade, A., Chan, A. W., King, M. T., et al. (2018). Guidelines for inclusion of patient-reported outcomes in clinical trial protocols: The SPIRIT-PRO Extension. *JAMA,**319*(5), 483. 29411037 10.1001/jama.2017.21903

[9] Giesinger, J. M., Wintner, L. M., Zabernigg, A., Gamper, E. M., Oberguggenberger, A. S., Sztankay, M. J., et al. (2014). Assessing quality of life on the day of chemotherapy administration underestimates patients’ true symptom burden. *BMC Cancer,**14*(1), Article 758. 25305067 10.1186/1471-2407-14-758 PMC4198707

[10] Shiroiwa, T., Hagiwara, Y., Taira, N., Kawahara, T., Konomura, K., Iwamoto, T., et al. (2022). Randomized controlled trial of paper-based at a hospital versus continual electronic patient-reported outcomes at home for metastatic cancer patients: Does electronic measurement at home detect patients’ health status in greater detail? *Medical Decision Making,**42*(1), 60–67. 33899589 10.1177/0272989X211010171

[11] Gwaltney, C. J., Shields, A. L., & Shiffman, S. (2008). Equivalence of electronic and paper-and-pencil administration of patient-reported outcome measures: A meta-analytic review. *Value in Health,**11*(2), 322–333. 18380645 10.1111/j.1524-4733.2007.00231.x

[12] Muehlhausen, W., Doll, H., Quadri, N., Fordham, B., O’Donohoe, P., Dogar, N., et al. (2015). Equivalence of electronic and paper administration of patient-reported outcome measures: A systematic review and meta-analysis of studies conducted between 2007 and 2013. *Health and Quality of Life Outcomes,**13*(1), Article 167. 26446159 10.1186/s12955-015-0362-x PMC4597451

[13] Mowlem, F. D., Elash, C. A., Dumais, K. M., Haenel, E., O’Donohoe, P., Olt, J., et al. (2024). Best practices for the electronic implementation and migration of patient-reported outcome measures. *Value in Health,**27*(1), 79–94. 37879401 10.1016/j.jval.2023.10.007

[14] U.S. Food and Drug Administration FDA. Patient-Focused Drug Development: Selecting, Developing, or Modifying Fit-for-Purpose Clinical Outcome Assessments [Internet]. 2022. Available from: https://www.fda.gov/regulatory-information/search-fda-guidance-documents/patient-focused-drug-development-selecting-developing-or-modifying-fit-purpose-clinical-outcome

[15] O’Donohoe, P., Reasner, D. S., Kovacs, S. M., Byrom, B., Eremenco, S., Barsdorf, A. I., et al. (2023). Updated recommendations on evidence needed to support measurement comparability among modes of data collection for patient-reported outcome measures: A good practices report of an ISPOR task force. *Value in Health,**26*(5), 623–633. 37121630 10.1016/j.jval.2023.01.001

[16] Yu, H., Yu, Q., Nie, Y., Xu, W., Pu, Y., Dai, W., et al. (2021). Data quality of longitudinally collected patient-reported outcomes after thoracic surgery: Comparison of paper- and web-based assessments. *Journal of Medical Internet Research,**23*(11), e28915. 34751657 10.2196/28915 PMC8663677

[17] Mercieca-Bebber, R., Palmer, M. J., Brundage, M., Calvert, M., Stockler, M. R., & King, M. T. (2016). Design, implementation and reporting strategies to reduce the instance and impact of missing patient-reported outcome (PRO) data: A systematic review. *British Medical Journal Open,**6*(6), Article e010938. 10.1136/bmjopen-2015-010938 PMC4916640 27311907

[18] Coons, S. J., Eremenco, S., Lundy, J. J., O’Donohoe, P., O’Gorman, H., & Malizia, W. (2015). Capturing patient-reported outcome (PRO) data electronically: The past, present, and promise of ePRO measurement in clinical trials. *Patient - Patient-Centered Outcomes Research,**8*(4), 301–309. 25300613 10.1007/s40271-014-0090-z PMC4529477

[19] Zbrozek, A., Hebert, J., Gogates, G., Thorell, R., Dell, C., Molsen, E., et al. (2013). Validation of electronic systems to collect patient-reported outcome (PRO) data—recommendations for clinical trial teams: Report of the ISPOR ePRO Systems Validation Good Research Practices Task Force. *Value in Health,**16*(4), 480–489. 23796281 10.1016/j.jval.2013.04.002

[20] Stone, A. A., Shiffman, S., Schwartz, J. E., Broderick, J. E., & Hufford, M. R. (2002). Patient non-compliance with paper diaries. *BMJ (Clinical Research Ed.),**324*(7347), 1193–1194. 12016186 10.1136/bmj.324.7347.1193 PMC111114

[21] Basch, E., Abernethy, A. P., Mullins, C. D., Reeve, B. B., Smith, M. L., Coons, S. J., et al. (2012). Recommendations for incorporating patient-reported outcomes into clinical comparative effectiveness research in adult oncology. *Journal of Clinical Oncology,**30*(34), 4249–4255. 23071244 10.1200/JCO.2012.42.5967

[22] European Medicines Agency. Guideline on computerised systems and electronic data in clinical trials [Internet]. 2023. Available from: https://www.ema.europa.eu/en/documents/regulatory-procedural-guideline/guideline-computerised-systems-and-electronic-data-clinical-trials_en.pdf

[23] Calvert, M. J., Cruz Rivera, S., Retzer, A., Hughes, S. E., Campbell, L., Molony-Oates, B., et al. (2022). Patient reported outcome assessment must be inclusive and equitable. *Nature Medicine,**28*(6), 1120–1124. 35513530 10.1038/s41591-022-01781-8

[24] Aiyegbusi, O. L., Cruz Rivera, S., Roydhouse, J., Kamudoni, P., Alder, Y., Anderson, N., et al. (2024). Recommendations to address respondent burden associated with patient-reported outcome assessment. *Nature Medicine,**29*, 1–10. 10.1038/s41591-024-02827-9 38424214

[25] Kyte, D., Draper, H., & Calvert, M. (2013). Patient-reported outcome alerts: Ethical and logistical considerations in clinical trials. *JAMA,**310*(12), 1229. 24065005 10.1001/jama.2013.277222

[26] Balitsky, A. K., Rayner, D., Britto, J., Lionel, A. C., Ginsberg, L., Cho, W., et al. (2024). Patient-reported outcome measures in cancer care: An updated systematic review and meta-analysis. *JAMA Network Open,**7*(8), e2424793. 39136947 10.1001/jamanetworkopen.2024.24793 PMC11322847

[27] Di Maio, M., Basch, E., Denis, F., Fallowfield, L. J., Ganz, P. A., Howell, D., et al. (2022). The role of patient-reported outcome measures in the continuum of cancer clinical care: ESMO clinical practice guideline. *Annals of Oncology,**33*(9), 878–892. 35462007 10.1016/j.annonc.2022.04.007

[28] Xia, Y., Guan, X., Zhu, W., Wang, Y., Shi, Z., & He, P. (2025). Effectiveness of symptom monitoring on electronic patient-reported outcomes (ePROs) among patients with lung cancer: A systematic review and meta-analysis. *NPJ Digital Medicine,**8*(1), 399. 40604235 10.1038/s41746-025-01812-x PMC12223247

[29] Madariaga A, Sánchez-Bayona R, Kasherman L, Estrada-Lorenzo JM, Manso L, Tolosa P, et al. (2025). Proactive assessment of patient reported outcomes in ovarian cancer studies: a systematic review and call for action in future studies. *International Journal of Gynecological Cancer,* 101864. 10.1136/ijgc-2024-005883 39379328

[30] Tricco, A. C., Lillie, E., Zarin, W., O’Brien, K. K., Colquhoun, H., Levac, D., et al. (2018). PRISMA extension for scoping reviews (PRISMA-ScR): Checklist and explanation. *Annals of Internal Medicine,**169*(7), 467–473. 30178033 10.7326/M18-0850

[31] Siegel, R. L., Miller, K. D., Fuchs, H. E., & Jemal, A. (2022). Cancer statistics, 2022. *CA: A Cancer Journal for Clinicians,**72*(1), 7–33. 35020204 10.3322/caac.21708

[32] Giesinger, J. M., Efficace, F., Aaronson, N., Calvert, M., Kyte, D., Cottone, F., et al. (2021). Past and current practice of patient-reported outcome measurement in randomized cancer clinical trials: A systematic review. *Value in Health,**24*(4), 585–591. 33840437 10.1016/j.jval.2020.11.004

[33] Lehmann, J., Krepper, D., Pe, M., Kuliś, D., Giesinger, J. M., Sztankay, M., et al. (2024). Data collection methods for patient-reported outcome measures in cancer randomised controlled trials: A protocol for a rapid scoping review. *British Medical Journal Open,**14*(9), Article e084935. 10.1136/bmjopen-2024-084935 PMC11409248 39260865

[34] DistillerSR [Internet]. DistillerSR Inc; 2025 [cited 2025 Sept 15]. Available from: https://www.distillersr.com

[35] Chan, A. W., Tetzlaff, J. M., Altman, D. G., Laupacis, A., Gøtzsche, P. C., Krleža-Jerić, K., et al. (2013). SPIRIT 2013 Statement: Defining Standard Protocol Items for Clinical Trials. *Annals of Internal Medicine,**158*(3), 200–207. 23295957 10.7326/0003-4819-158-3-201302050-00583 PMC5114123

[36] Moher D, Hopewell S, Schulz KF, Montori V, Gotzsche PC, Devereaux PJ, et al. (2010). CONSORT 2010 Explanation and Elaboration: updated guidelines for reporting parallel group randomised trials. *BMJ*, *340*(mar23 1), c869–c869. 10.1136/bmj.c869 PMC2844943 20332511

[37] R Core Team. (2021). R: A language and environment for statistical computing [Internet]. Vienna, Austria: R Foundation for Statistical Computing. Available from: https://www.R-project.org/

[38] Bylicki, O., Gan, H. K., Joly, F., Maillet, D., You, B., & Péron, J. (2015). Poor patient-reported outcomes reporting according to CONSORT guidelines in randomized clinical trials evaluating systemic cancer therapy. *Annals of Oncology,**26*(1), 231–237. 25355720 10.1093/annonc/mdu489

[39] Efficace, F., Giesinger, J. M., Cella, D., Cottone, F., Sparano, F., Vignetti, M., et al. (2021). Investigating trends in the quality of reporting of patient-reported outcomes in oncology over time: Analysis of 631 randomized controlled trials published between 2004 and 2019. *Value in Health,**24*(12), 1715–1719. 34838268 10.1016/j.jval.2021.06.003

[40] Chan, A. W., & Hróbjartsson, A. (2018). Promoting public access to clinical trial protocols: Challenges and recommendations. *Trials,**19*(1), Article 116. 29454390 10.1186/s13063-018-2510-1 PMC5816550

[41] Hopewell, S., Chan, A. W., Collins, G. S., Hróbjartsson, A., Moher, D., Schulz, K. F., et al. (2025). CONSORT 2025 statement: Updated guideline for reporting randomized trials. *Nature Medicine,**31*(6), 1776–1783. 40229553 10.1038/s41591-025-03635-5

[42] José, N., & Langel, K. (2010). ePRO vs. Paper. *Applied Clinical Trials,**19*, 68–74.

[43] Mowlem, F. D., Tenaerts, P., Gwaltney, C., & Oakley-Girvan, I. (2022). Regulatory acceptance of patient-reported outcome (PRO) data from bring-your-own-device (BYOD) solutions to support medical product labeling claims: Let’s share the success stories to move the industry forward. *Therapeutic Innovation & Regulatory Science,**56*(4), 531–535. 35534774 10.1007/s43441-022-00412-1 PMC9084261

[44] Aiyegbusi, O. L., Cruz Rivera, S., Kamudoni, P., Anderson, N., Collis, P., Denniston, A. K., et al. (2024). Recommendations to promote equity, diversity and inclusion in decentralized clinical trials. *Nature Medicine,**30*(11), 3075–3084. 39472759 10.1038/s41591-024-03323-w

[45] Kim, J., Singh, H., Ayalew, K., Borror, K., Campbell, M., Johnson, L. L., et al. (2018). Use of PRO measures to inform tolerability in oncology trials: Implications for clinical review, IND safety reporting, and clinical site inspections. *Clinical Cancer Research,**24*(8), 1780–1784. 29237718 10.1158/1078-0432.CCR-17-2555

[46] Basch, E., Wood, W. A., Schrag, D., Sima, C. S., Shaw, M., Rogak, L. J., et al. (2016). Feasibility and clinical impact of sharing patient-reported symptom toxicities and performance status with clinical investigators during a phase 2 cancer treatment trial. *Clinical Trials,**13*(3), 331–337. 26542025 10.1177/1740774515615540 PMC5228492

[47] Kyte, D., Ives, J., Draper, H., & Calvert, M. (2016). Management of patient-reported outcome (PRO) alerts in clinical trials: A cross sectional survey. *PLoS ONE,**11*(1), e0144658. 26785084 10.1371/journal.pone.0144658 PMC4718453

[48] Lehmann J. (2025). After the trial is too late: The case for more active use of patient-reported outcomes in oncology studies. *Applied Clinical Trials*, *34*(4).

[49] Hubel, N. J., Vorbach, S. M., De Ligt, K. M., Rathgeber, I. S., Beyer, K., Wintner, L. M., et al. (2025). Sustainability and time trends in electronic patient-reported outcome assessment in routine cancer care: Systematic scoping review and follow-up survey. *Journal of Medical Internet Research,**25*(27), e69398. 10.2196/69398 PMC12064961 40280556

