# Supplementary material for: Assessing the assessment: a scoping review of the mode of patient-reported outcome assessment in solid cancer clinical trials
Source: Qual Life Res. 2026 Mar 1;35(4):80. doi: 10.1007/s11136-026-04179-y (PMC12950655; doi:10.1007/s11136-026-04179-y)
Supplement: Supplementary file 1 — Supplementary Material 1 [file 11136_2026_4179_MOESM1_ESM.pdf]

Table S1: Active review of PRO results

Trial ID/NCT	Author	Relevant section from the protocol
NCT02422615	G Jerusalem, TE Delea, M Martin, M De Laurentiis, A Nusch, JT Beck, A Chan, SA Im, P Neven, A Lonshteyn, D Chandiwana, B Lanoue, PA Fasching	*"Completed questionnaires, including both responses to the questions and any unsolicited comments written by the patient, must be reviewed and assessed by the investigator before the clinical examination for responses which may indicate potential AEs or SAEs. This review should be documented in study source records."*
NCT02131064	SA Hurvitz, M Martin, KH Jung, CS Huang, N Harbeck, V Valero, D Stroyakovskiy, H Wildiers, M Campone, JF Boileau, PA Fasching, K Afenjar, G Spera, V Lopez-Valverde, C Song, P Trask, T Boulet, JA Sparano, WF Symmans, AM Thompson, D Slamon	*"Adverse event reports will not be derived from PRO data. However, if any patient responses suggestive of a possible adverse event are identified during site review of the PRO questionnaires, site staff will alert the investigator, who will determine if the criteria for an adverse event have been met and will document the outcome of this assessment in the patient's medical record per site practice. If the event meets the criteria for an adverse event, it will be reported on the adverse event eCRF."*
NCT02278120	YS Lu, SA Im, M Colleoni, F Franke, A Bardia, F Cardoso, N Harbeck, S Hurvitz, L Chow, J Sohn, KS Lee, S Campos-Gomez, R Villanueva Vazquez, KH Jung, KG Babu, P Wheatley-Price, M De Laurentiis, YH Im, S Kuemmel, N El-Saghir, R O'Regan, C Gasch, N Solovieff, C Wang, Y Wang, A Chakravartty, Y Ji, D Tripathy	*"Completed questionnaires, including both responses to the questions and any unsolicited comments written by the patient, must be reviewed and assessed by the investigator before the clinical examination for responses which may indicate potential AEs or SAEs. This review should be documented in study source records."*
NCT01966471	IE Krop, SA Im, C Barrios, H Bonnefoi, J Gralow, M Toi, PA Ellis, L Gianni, SM Swain, YH Im, M De Laurentiis, Z Nowecki, CS Huang, L Fehrenbacher, Y Ito, J Shah, T Boulet, H Liu, H Macharia, P Trask, C Song, EP Winer, N Harbeck	*"AE reports will not be derived from PRO data. However, if any patient responses suggestive of a possible AE are identified during site review of the PRO questionnaires, site staff will alert the investigator, who will determine if the criteria for an AE have been met and will document the outcome of this assessment in the patient's medical record per site practice. If the event meets the criteria for an AE, it will be reported on the Adverse Event eCRF."*
NCT02366143	M Reck, T Wehler, F Orlandi, N Nogami, C Barone, D Moro-Sibilot, M Shtivelband, JL González Larriba, J Rothenstein, M Früh, W Yu, Y Deng, S Coleman, G Shankar, H Patel, C Kelsch, A Lee, E Piault, MA Socinski	*"Adverse event reports will not be derived from PRO data, and safety analyses will not be performed using PRO data. However, if any PRO responses suggestive of a possible adverse event are identified during site review of the PRO data, the investigator will determine whether the criteria for an adverse event have been met and, if so, will report the event on the Adverse Event eCRF."*
NCT02115282	TJ Ballinger, HS Marques, G Xue, R Hoffman, C Gatsonis, F Zhao, KD Miller, J Sparano, RM Connolly	*"Clinicians will be instructed to review PRO-CTCAE items after clinician toxicity ratings have been completed to identify any patient-reported symptoms and toxicities that warrant clinical attention. Supportive care measures to manage treatment toxicities are described in Section 5.5."*
